# Vaginal Evisceration: An Unexpected Complication of Conization

**DOI:** 10.1155/2014/983682

**Published:** 2014-11-19

**Authors:** Ali Ghassani, Benoit Andre, Caroline Simon-Toulza, Yann Tanguy le Gac, Alejandra Martinez, Fabien Vidal

**Affiliations:** ^1^CHU Toulouse, Pole de Gynécologie Obstétrique, Hôpital Paule de Viguier, 31059 Toulouse, France; ^2^Surgery Department, Institut Claudius Regaud, 20-24 rue du pont Saint Pierre, 31052 Toulouse, France; ^3^Hôpital Paule de Viguier-CHU Toulouse, Service de Chirurgie Gynécologique, Pole de Gynécologie Obstétrique, 31059 Toulouse Cedex 9, France

## Abstract

*Background.* Large loop excision of the transformation zone (LLETZ) is routinely performed for the management of high grade intracervical neoplasia (CIN). Several uncommon complications have been described, including postoperative peritonitis, pseudoaneurysm of uterine artery, and bowel fistula. We report a unique case of postoperative vaginal evisceration and the subsequent management. *Case.* A 73-years-old woman underwent LLETZ for high grade CIN. On postoperative day 3, she was admitted for small bowel evisceration through the vagina. Surgical management was based on combined laparoscopic and transvaginal approach and consisted in bowel inspection and reinstatement, peritoneal washing, and dehiscence repair. *Conclusions.* Vaginal evisceration is a rare but potentially serious complication of pelvic surgery. This case report is to make clinicians aware of such complication following LLETZ and its management.

## 1. Introduction

Conization is the mainstay of treatment for high grade cervical intraepithelial neoplasia (CIN). Three different excisional methods (cold knife conization, loop electrosurgical excision, and laser conization) are available and, to date, none of them seem superior to another [1]. Large loop excision of the transformation zone (LLETZ) is a common surgical procedure using a small wire loop and electrical current, with recognized complications such as bleeding, infection, and postoperative cervical stenosis [2]. It is also associated with poorer obstetrical outcomes, including increased rates of preterm delivery and perinatal mortality [2]. Most procedures are performed under local anesthesia with ambulatory care.

Herein we report the first case to our knowledge of acute vaginal evisceration following LLETZ.

## 2. Observation

A 73-years-old woman was referred to our institution for the management of high grade intraepithelial neoplasia confirmed by colposcopy-guided biopsy. LLETZ procedure was performed under general anesthesia and ended with monopolar coagulation of the posterior part of the resection because of arterial and venous bleeding. A vaginal mesh was left in place and removed before the patient was discharged, on the day of the surgery.

**Figure 1:**
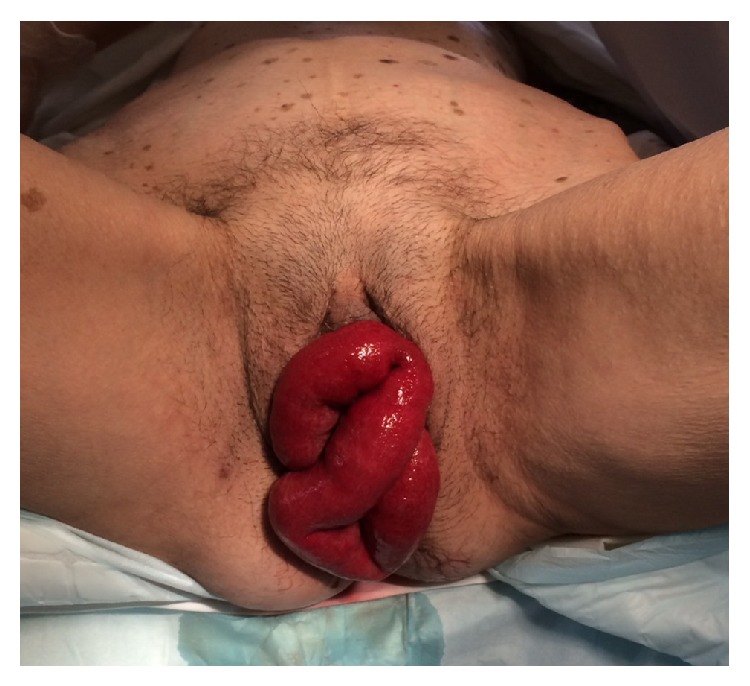
Small bowel evisceration through the vagina.

Three days after surgery, the patient was admitted in the emergency department for bulging vagina and bowel obstructive syndrome. She had no history of postoperative bleeding or pain. Clinical examination revealed small bowel evisceration through the vagina. Bowel loops were edematous but viable (Figure 1).

**Figure 2:**
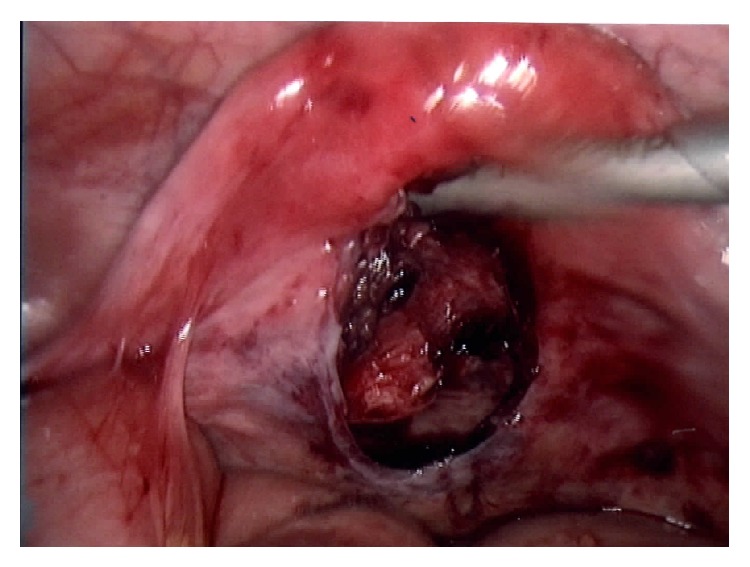
Vaginal wall defect (laparoscopic view).

Evisceration was managed by a combined laparoscopic and vaginal approach. Small bowel reinstatement was performed vaginally with laparoscopic guidance and revealed a large defect in the vaginal posterior wall next to the site of LLETZ (Figure 2). Laparoscopic evaluation found neither bowel abnormalities nor any peritonitis.

**Figure 3:**
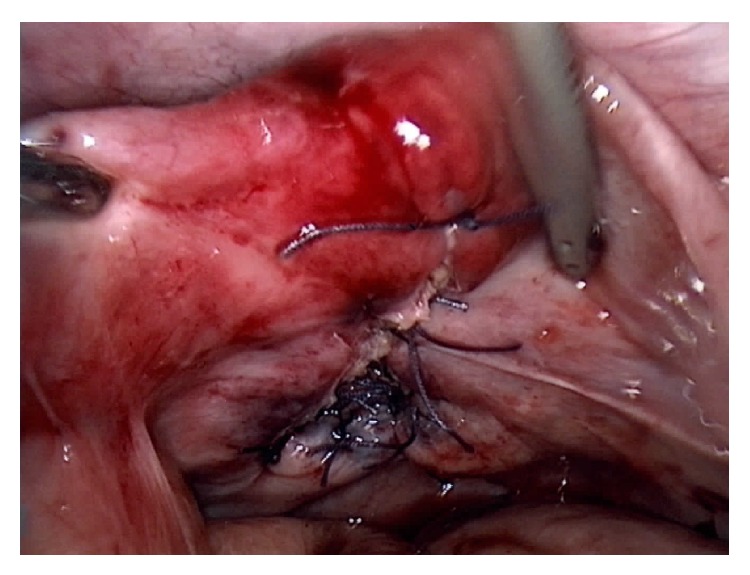
Laparoscopic repair of the defect involving Vicryl simple suture.

Treatment consisted in peroperative antibiotic prophylaxis (amoxicillin plus clavulanic acid 2 g), peritoneal washing, and vaginal repair with interrupted laparoscopic Vicryl sutures (Figure 3). Procedure duration was 90 min.

The patient recovered uneventfully and was discharged on 4th postoperative day. Delayed follow-up was normal.

## 3. Discussion

In this report, we introduce the first published case of vaginal evisceration following LLETZ. It was managed by a combined vaginal and laparoscopic approach and consisted in bowel reinstatement, peritoneal washing, and vaginal repair.

Vaginal evisceration is a rare but potentially life threatening complication. Its incidence after any type of pelvic surgery is 0.03% [3, 4]. Major risk factors are postmenopausal state, increased abdominal pressure, and hysterectomy [5]. Among patients who undergo hysterectomy, laparoscopic route is associated with a higher incidence of cuff dehiscence, compared to transvaginal and abdominal procedures [6].

LLETZ is a common surgical procedure for the management of CIN with recognized complications. Several uncommon complications of conization have been described including fistula formation [7], intestinal occlusion [8], intra-abdominal hemorrhage [9], pseudoaneurysm of uterine artery [10], peritonitis [11], retroperitoneal hematoma [12], uterine avulsion [13], and extrapelvic abscesses [14].

Vaginal evisceration following LLETZ is quite surprising since the vagina is not concerned by the resection. Etiologic mechanism may thus involve an unrecognized iatrogenic posterior colpotomy or necrosis of the posterior vaginal fornix due to monopolar coagulation. Similar event has been previously reported by Varras et al. [11] but led to infectious peritonitis rather than evisceration.

Morbidity associated with vaginal evisceration is high: 15% of patients develop postoperative complications and 20% require bowel resection [15]. Delay in diagnosis and treatment increases the risk of bowel infarction. Hence its management is a medical emergency.

Surgery is the mainstay of treatment. Concomitant intravenous administration of antibiotics is recommended due to bowel extraperitoneal exposure [11, 15–17]. Several routes of surgery have been described: vaginal, abdominal, or combined [5, 17, 18]. Regarding abdominal route, former laparotomy approach has been replaced by laparoscopy whenever applicable [15]. According to a review focusing on vaginal evisceration following hysterectomy, surgical approaches are mostly vaginal or abdominal while only 15% of the patients benefit from a combined approach [5]. We strongly recommend the use of such combined surgery that allows excluding associated and unrecognized bowel injuries and washing properly the peritoneal cavity [19–21]. It should be systematically performed in patients who might have poor tissue quality (postmenopausal, gynecologic malignancies, history of pelvic radiation therapy).

In most cases, the surgical management of vaginal defect consists in a primary repair involving simple suture. Some authors have also described techniques using synthetic mesh or omental flap to strengthen vaginal repair [15, 22, 23].

The prevention of such complication is another important issue, although the etiologic mechanism remains unclear in our case. Nevertheless, caution should be paid in the use of monopolar coagulation, particularly in atrophic tissues. Bipolar energy or hemostatic sutures should be preferred for persistent and severe bleeding. To avoid unintentional colpotomy, a soft pulling should be exerted on the cervix during LLETZ. Last, whenever a peroperative complication such as hemorrhage occurs, the use of vaginal retractors may improve exposure and subsequent management. Indeed, vaginal dehiscence can be managed with minimal morbidity if recognized immediately.

## 4. Conclusion

Vaginal evisceration is a rare but potentially serious complication of pelvic surgery. Most cases were described after hysterectomy. We introduce here the first report to our knowledge of such complication after conization.

Vaginal evisceration is a surgical emergency, since delay in treatment exposes to increased morbimortality. Our management was based on a combined laparoscopic and transvaginal approach leading to favorable outcomes. Although our single case report does not provide a strong level of evidence, we highly recommend this surgical attitude for the management of such complication.
